# Management Practices During Perinatal Respiratory Transition of Very Premature Infants

**DOI:** 10.3389/fped.2022.862038

**Published:** 2022-05-10

**Authors:** Mikko Hallman, Eveliina Ronkainen, Timo V. Saarela, Riitta H. Marttila

**Affiliations:** ^1^PEDEGO Research Unit, MRC Oulu, University of Oulu, Oulu, Finland; ^2^Department of Children and Adolescents, Oulu University Hospital, Oulu, Finland

**Keywords:** respiratory distress syndrome, spontaneous premature birth, prenatal steroid, surfactant therapy, ductus arteriosus, paracetamol, persistence of pulmonary hypertension, inhaled nitric oxide

## Abstract

The present review considers some controversial management practices during extremely premature perinatal transition. We focus on perinatal prevention and treatment of respiratory distress syndrome (RDS) in immature infants. New concerns regarding antenatal corticosteroid management have been raised. Many fetuses are only exposed to potential adverse effects of the drug. Hence, the formulation and the dosage may need to be modified. Another challenge is to increase the fraction of the high-risk fetuses that benefit from the drug and to minimize the harmful effects of the drug. On the other hand, boosting anti-inflammatory and anti-microbial properties of surfactant requires further attention. Techniques of prophylactic surfactant administration to extremely immature infants at birth may be further refined. Also, new findings suggest that prophylactic treatment of patent ductus arteriosus (PDA) of a high-risk population rather than later selective closure of PDA may be preferred. The TREOCAPA trial (Prophylactic treatment of the ductus arteriosus in preterm infants by acetaminophen) evaluates, whether early intravenous paracetamol decreases the serious cardiorespiratory consequences following extremely premature birth. Lastly, is inhaled nitric oxide (iNO) used in excess? According to current evidence, iNO treatment of uncomplicated RDS is not indicated. Considerably less than 10% of all very premature infants are affected by early persistence of pulmonary hypertension (PPHN). According to observational studies, effective ventilation combined with early iNO treatment are effective in management of this previously fatal disease. PPHN is associated with prolonged rupture of fetal membranes and birth asphyxia. The lipopolysaccharide (LPS)-induced immunotolerance and hypoxia-reperfusion-induced oxidant stress may inactivate NO-synthetases in pulmonary arterioles and terminal airways. Prospective trials on iNO in the management of PPHN are indicated. Other pulmonary vasodilators may be considered as comparison drugs or adjunctive drugs. The multidisciplinary challenge is to understand the regulation of pregnancy duration and the factors participating the onset of extremely premature preterm deliveries and respiratory adaptation. Basic research aims to identify deficiencies in maternal and fetal tissues that predispose to very preterm births and deteriorate the respiratory adaptation of immature infants. Better understanding on causes and prevention of extremely preterm births would eventually provide effective antenatal and neonatal management practices required for the intact survival.

## Introduction

The preterm birth (PTB) before full 37 weeks duration of pregnancy is the main underlying cause of neonatal deaths, and the predominant risk factor of the respiratory distress syndrome (RDS). Although the introduction of antenatal steroid, surfactant supplementation and other management practices have decreased both the incidence and the fatality of RDS, major morbidities continue to threaten the intact survival (1). The countries with middle and low incomes still do not have the resources to organize the neonatal special care for immature infants with RDS. However, the rate of premature births and the chronic morbidities due to prematurity also remains high in wealthier countries (2). Introduction of surfactant supplementation and the common use of antenatal glucocorticoid therapy led to survival of PTB infants and those born at very low gestational ages (VLGA; <32 weeks of pregnancy). Increasing numbers of infants born at extremely low gestational age (ELGA: <28 weeks of pregnancy) survive with advancing management practices (3). Extremely preterm infants with RDS require supplemental oxygen and ventilation for treatment of atelectasis and lung edema. In addition, they have structural immaturity of the lungs and hemodynamic problems, disturbing the gas exchange of lungs (4, 5). The treatment practices of RDS are still evolving and continue to be controversial (6).

The duration of pregnancy is the main factor influencing the fetal maturity, including the differentiation of surfactant which has dominant impact on RDS. Spontaneous premature births (SPTB) associate with histologic or clinical chorioamnionitis (CA) and prolonged rupture of fetal membranes (PPROM) (7). Intrauterine growth restriction (IUGR), associating with preeclampsia, twin or multiple pregnancies, and many other rare conditions influence the neonatal disease profile. Histologic CA without clinical maternal or fetal infection accelerates fetal surfactant maturity, whereas fetal infection, clinical CA, asphyxia, growth restriction, and, above all, extremely premature births may influence the risk of severe RDS and complicate the management of respiratory distress (8–10). Minimization of postnatal supplementary oxygen and mechanical ventilation requirements (11) protect the lung and other organs against inflammatory and oxidant injury. However, antenatal risk factors, particularly structural immaturity, complicate the management of RDS and play a major role in the pathogenesis of long term morbidities, particularly bronchopulmonary dysplasia (BPD) (12). The new BPD was considered as a disease that gradually developed after the first days of life. This drew the attention to the management one to a few days *after* the birth. However, the importance of the management of early perinatal transition is essential.

During the 1990s, both antenatal corticosteroid (ACS) therapy for acceleration of the organ maturity and surfactant supplementation for treatment of RDS became common management policy of very premature perinatal transition (13). According to meta-analyses of randomized trials, certain treatment practices, such as caffeine therapy, vitamin A, low dose of steroid during early neonatal period, less invasive surfactant therapy, and new, mostly non-invasive ventilation practices have increased the neonatal survival without serious morbidities, particularly BPD (12, 14). However, according to population-wide data, there has only been modest, if any, increase in survival without serious morbidities during the present millennium (15, 16).

Previously, nearly all VLGA infants used to receive surfactant shortly after birth. Recently, surfactant treatment has become more selective. The current aim to practice less invasive surfactant administration does not require intubation and improves survival without BPD (17–19). The objective is to decrease intubation and mechanical ventilation, and to increase continuous distending pressure ventilation. Meanwhile, pharmacological or surgical closure of patent ductus arteriosus (PDA) has failed to increase the survival of VLGA or ELGA infants without BPD. According to current recommendations, conservative treatment of PDA, i.e., continuous distending pressures and limitation of liquid overload, is often preferred during the first week, and the medicinal closure of PDA becomes indicated after that (6). The other end of the spectrum of RDS-associated cardiopulmonary disorders is the early persistent pulmonary hypertension (PPHN), also called the persistence of fetal circulation (PFC). According to meta-analysis of randomized trials, inhaled nitric oxide (iNO) treatment of VLGA infants did not improve the outcome. The indications of iNO treatment in these trials varied from mild to severe respiratory distress (20).

Very premature birth often triggers an inflammatory and oxidant storm spreading to multiple organs. In many cases, it is a continuation of the fetal inflammatory syndrome (12, 21, 22). The premature labor and delivery and the treatment of immature infant immediately after birth is a successive collaboration of two different teams. Many mothers with spontaneous premature delivery and their fetuses at birth are basically healthy, but for mostly unknown causes, the onset of premature labor results in a stressful neonatal adaptation that often dramatically alters the life of the child and the family, thereby needing support and guidance as an important part of the team. As such, prematurity and the associated pre- and antenatal factors modify the incidence and severity of RDS (23). Inflammation and toxic oxidants may both be early precursors of premature birth and mediators that increase the risk of multiple consequences of extremely premature births (10, 21, 24).

Research on organ maturation and on regulation of the length of pregnancy may lead to new drug discoveries for the prevention and therapy of RDS. The new candidates include stem cell, exosome, and growth factor therapies. Systematic research on selective treatments toward increasing the defense functions of premature children and very preterm pregnancies is crucial. Progress in the management practices of early perinatal transition will decrease the risk of severe RDS and improve the early recovery from cardiopulmonary distress. This is expected to decrease the risk of BPD, severe multiorgan consequences, and to contribute toward improving neurosensory outcome. In present review, we focus on a few key therapies that are available and require further development.

## Newborn-Oriented Research Towards Prolonging Very Short Pregnancies

### Preterm Births Linked to RDS

The global risk of the premature birth ranges from 5 to 20% with a mean risk of approximately 11%, or about 15 million PTBs each year. The influence of premature birth to the loss of disability-adjusted life years amounts to 80 million each year (25). Multiple diseases or insults affecting the mother, fetus, or placenta result in elective premature births, accounting for 30% of all preterm births. Of them, severe preeclampsia is common, involving nearly half of elective preterm births. The indications of early delivery include serious fetal circulatory stress due to an increase in resistance of umbilical circulation, fetal growth restriction associated with deficient placental oxygen and nutrient intake, and serious maternal diseases.

The most common cause of prematurity is SPTB (24). The SPTBs include about 70% of all preterm births, i.e., nearly 4% of all births in Nordic countries and about 8% of all births globally (26, 27).

According to the reported incidence figures, there are nearly three million cases of RDS annually (28), and the gestation adjusted mortality of RDS is several-fold higher than in the controls (29). Among the ELGA infants born between 22 and 27 weeks, the incidence of RDS is above 50%. The deaths of the most immature infants with the diagnosis of RDS or respiratory failure are due to multiple diseases and deficiencies, and most of them occur during the first week of life. Many survivors requiring prolonged ventilation and supplemental oxygen will end up having the diagnosis of BPD. The new diagnosis of BPD still associates with the severity of RDS. Besides the surfactant deficiency, the immaturity is generalized. It affects structures and functions required for cardio-pulmonary adaptation and extends to deficiencies in the defense functions (12).

Most of the preterm infants, born at 32–36 week, do not develop RDS, apparently due to advanced structural maturity and genetic predisposition to surfactant maturity (23), whereas RDS is common among the VLGA infants. However, ACS treatment, early surfactant therapy, and mostly non-invasive continuous distending pressure ventilation techniques mitigate the symptoms and improve the outcome. In the era of ACS and early surfactant therapy, antenatal prediction of the risk of RDS [lecithin/sphingomyelin (L/S) ratio, Lung Profile or Lamellar bodies] before VLGA births is not indicated.

### Spontaneous Preterm Birth in Very Premature Infants

In most cases of SPTB, the labor starts early, without identifiable cause and progresses regardless of the tocolytic therapies. This is by far the most common cause of births at weeks 22–25 (7). PPROM predisposes or precedes very preterm SPTB in nearly 30% of all cases. Excessive uterine distension, typically in multiple pregnancies, increases the risk of SPTB. Smoking, alcoholism, the use of narcotic or euphoric drugs, and low socioeconomic status are associated with increase in the risk of SPTB. Very young age of mothers (<18 years), short stature, very high pre-pregnancy body mass index (BMI > 40), and stressful work in standing position are reported risk factors. Previous SPTB is associated with a 3 to 5-fold increase in the recurrence rate being a prominent risk factor of SPTB (7, 30).

Chorioamnionitis often presents as a non-infectious inflammation in fetal membranes. Intrauterine infection/ inflammation spreads from the ascending route via maternal blood or through the Fallopian tube, and it may manifest as clinical CA with prominent maternal and fetal symptoms. CA is present in 70–95% extremely premature (gestation < 28 weeks) and in 5–15% of term births (31). Antibiotic prophylaxis delays SPTB in PPROM, decreasing the need for surfactant and supplemental oxygen therapies (32). By mid-pregnancy, certain biomarkers of inflammation tend to increase, and this increase edges even higher in premature pregnancies compared to full length pregnancies. However, both the sensitivity and predictive value of positive results in imminent early premature birth are rather low even for the most accurate vaginal biomarkers, fetal fibronectin, glycosylated IGFBP-1, or β-microglobulin (33).

Hereditary factors play significant roles in predisposing to SPTB (30), and many gene variants influence the susceptibility (34). According to epidemiologic studies, the maternal genome contributes 15–30% of the SPTB risk, and the contribution of the fetal genome is 7–14%. The overall genetic risk estimate of SPTB is 25–40%. Human pregnancy appears to be linked to the recent evolution of bipedalism, including having a narrow pelvis, a burst in cognitive capacity, and increase in brain volume and head size (35). The length of the human pregnancy has an exceptionally large variation. Even the surfactant system is often capable of supporting the lung function several weeks before term. The medical practices have virtually eliminated the obstructed labor syndrome and postmature deliveries. In contrast, effective prevention of SPTB has failed thus far.

#### Molecular Medicine Linked to Epidemiology: Toward Understanding Causes of SPTB

Several tissues may harbor “the clock” determining the pregnancy duration. Proposed tissues include those of the uterus, placenta, fetal membranes, and other intrauterine tissues, along with endocrine and neuronal tissues. The increase in stress and inflammation generally increases the risk of premature labor and concomitantly influences fetal maturity (24, 36). However, the immune system is complex, and individual components are often multifunctional, compartmentalized, and interactive, complicating the use of biological drugs. The studies involving the whole genome, exome, proteome, and other omics studies linked to epidemiologic information provide valuable hypothesis-free data on possible causes of SPTB. Some of the maternal candidate genes, pointed out by the genomic studies, are already functionally active during the decidualization (*WNT-4*, *HSPA1L*, and others) (34, 37–39). However, the tissues and mediators that signal the onset of labor have not been accurately identified (24). An active research focus is the highly organized heterologous structures of placenta, fetal membranes, decidual tissue, and the maternal blood perfusing the intervillous space. They are intimately connected to the fetus and the labor-producing uterine corpus and cervix.

A recent prospective study compared the proteomes of placentas from very preterm SPTB to the proteomes of placentas from STB and those from elective very preterm deliveries (EPTB) (40). Among some 10,000 identified proteins, six were distinctly different in SPTB placentas. The exon sequences of genes encoding these six proteins were studied for rare damaging variants in both mothers and fetuses suffering from recurrent SPTBs, and only the *SERPINA1* gene, which encodes alpha-1 antitrypsin (AAT), contained one of the three rare variants in some individuals affected by SPTB. AAT was localized and expressed in trophoblasts. It localizes within cytoplasmic granules of syncytiotrophoblasts and is secreted to placental intervillous space, potentially contributing to the known increase in serum AAT concentration during pregnancy (BR41a,). AAT is apparently also secreted from the decidua trophoblasts to the fibrinoid tissue. In addition, within the decidua fibrinoid, it is associated with specific collagens and is also located around fetal fibronectin (Fe-FN1). *SERPINA1* knockdown in human trophoblast cell line-augmented genes regulating the inflammatory response, inhibited the formation and organization of the extracellular matrix components and increased synthesis of Fe-FN1, a biomarker predicting SPTB (40). These findings led us to propose that AAT/*SERPINA1* within the internal lining of the uterus contributes to the maintenance of pregnancy to term. Both the AAT content and *SERPINA1* expression are significantly decreased in the placenta compared to those in STB and EPTB placentas (40). According to *SERPINA1* silencing experiment, AAT-deficiency decreases the anti-inflammatory and antiprotease capacities and the capacity to maintain the structural integrity of extracellular matrix lining between the uterus and the placenta/fetal membranes.

Alpha-1 antitrypsin treatment delays the progress hereditary lung disease due to rare damaging variants of *SERPINA1*. AAT deficiency is also evident in the airways of RDS infants developing BPD (42). In addition, a therapeutic trial of AAT supplementation of premature infants decreased the risk of pulmonary hemorrhage (43). Further studies are required to explore the potential benefits of AAT in the management of very premature infants with respiratory distress. Studies are in progress to further investigate the roles of AAT in SPTB and, among others, to investigate whether the supplementation of AAT has a capacity to prevent induced preterm birth in experimental SPTB. Despite multiple failures to prevent SPTB during past 80 years, recent findings point out that systematic research may eventually provide new tools for prevention of very premature births.

In humans and great apes, unlike other mammals, the decrease in progesterone (P4) secretion does not induce labor. Instead, P4 secretion from villous syncytiotrophoblasts continues to increase during the pregnancy and labor. P4 supplementation has a capacity to prevent SPTB, but only in rare cases of the short cervix syndrome (44). The activity of the progesterone receptor (PGR) may have important role in the induction of human SPTB. *PGR*, a nuclear transcription factor, is prominently expressed in uterus. Human PGR-B mediates the anti-inflammatory effect of P4, silencing the uterine musculature, while PGR-A isoform silences the effect of PGR-B. In a uterine muscle cell line, the labor-inducing contractile response of P4 is promoted by the inflammatory mediator (IL1β) as it increases the expression of *PGR-A* and decreases that of *PGR-B* (45). Certain variants that influence the expression of *PGR* are unique in the modern human species (46).

Several anti-inflammatory agents, as inhibitors of proinflammatory mediators, have a potential in prevention of SPTB. These drugs have multiple effects, and the inhibitors of inflammation may perturb the balance of perinatal transition and cause serious adverse effects to the fetus and newborn. A desirable drug both prolongs the duration of a very short pregnancy and promotes the respiratory adaptation without adversely influencing growth or critical differentiation of the brain and other organs. Investigation on causes, consequences, and prevention of very premature birth is becoming a lucrative area of neonatal-perinatal research.

## Major Therapies That Continue to Develop or May Still Be Controversial

### Antenatal Corticosteroid Treatment

Potential adverse long term consequences of ACS in imminent preterm birth must be weighed against the benefits of advancing the fetal maturity and prevention of serious diseases: survival without RDS, intraventricular hemorrhage (IVH), necrotizing enterocolitis (NEC), and possibly BPD. Unfortunately, the follow-up results of ACS therapy in young adults are based upon limited availability of data from randomized trials as they mostly originate from retrospective population-based registers. Increased risk of CP is reported after multiple repeat doses of ACS (47). Some cohort studies later in childhood and young adults have found evidence on neuropsychological and cognitive disorders in children exposed to ACS (48, 49), whereas others fail to observe similar abnormalities (50, 51). In randomized trials using antenatal steroids in threatened very premature birth, the continuation of pregnancy to term has occurred in 15–60% of all fetuses exposed to ACS in threatened premature birth (52). Thus, a significant proportion of these presumably high-risk fetuses were only exposed to potentially harmful effects of the steroid (49). However, the adverse effects found in the cohort studies of ACS-exposed children may also be due to pregnancy complications or circumstances unrelated to ACS.

Antenatal fluorinated steroids, betamethasone (BM), or dexamethasone (DX) pass the uterine barrier and dose-dependently decrease the growth of the preterm fetus (52). Growth restriction at birth is a risk factor associating with adverse cardiopulmonary effects during childhood and in later life (53, 54). Besides retardation of the fetal growth, the ACS programming associates with long term morbidities, including the risk of cognitive and neuropsychiatric disorders, chronic lung disease, hypertension, diabetes, coronary disease, and early death (55).

The debate whether to repeat the ACS dosage continues as more data accumulates (52). According to meta-analysis, the only observed beneficial effect of the repeat ACS dosage is the early treatment requirement of respiratory distress. However, this may not be clinically relevant. The latest European consensus report for RDS treatment states that the well-intended repeat of ACS treatment is potentially harmful in the long term (6). Obtaining the maximal benefit and minimizing the adverse effects of ACS is in part dependent on how accurately the drug can be targeted. The benefit of ACS may be optimal when the fetus is born before 32 weeks of pregnancy and 1–7 days after the first ACS dose. The high precision of the timing of ACS is a challenge since the biomarkers predicting the time of the delivery are not very accurate, and the efficacy of the tocolytic therapies is variable.

Another evidence suggests that even the single ACS dosage used may be excessive. Liggins and Howie used a single dosage of BM (6 mg BM-phosphate, 6 mg BM-acetate × 2 q. 24 h intramuscularly, i.m.) (56), and in the US trial, a single dosage of DX (5–6 mg × 4, q. 12 h) was given (57). Similar ACS dosages have been given in most subsequent single dosage studies. According to a recent pharmacodynamic study utilizing both maternal and cord blood levels of BM, injections of 5.7 mg i.m. 24 h apart may provide sufficient fetal BM for optimal pharmacologic effect (58). In twin pregnancies, the maternal clearance rate of BM was increased by a mean of 28% (58).

**FIGURE 1:**
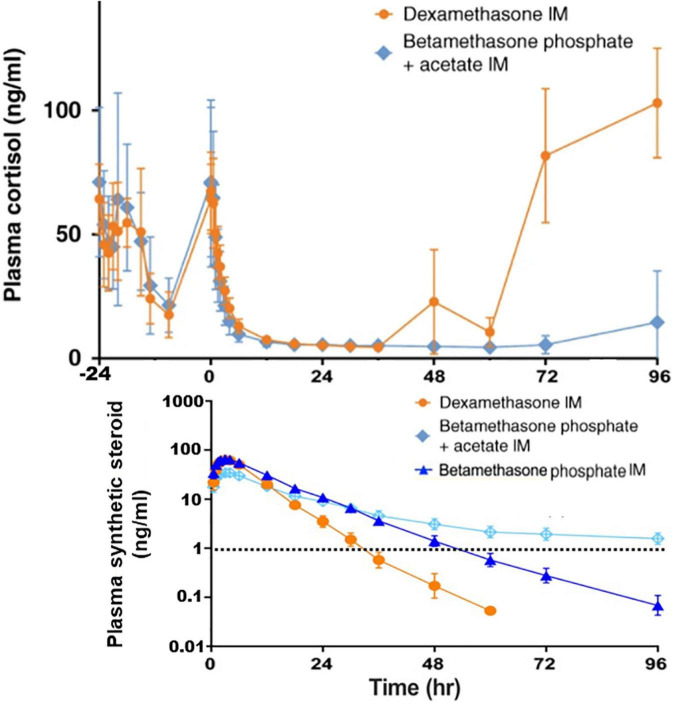
Measurements of plasma concentrations of cortisol and the synthetic steroids in non-pregnant female subjects. Cortisol concentrations are shown starting 24 h before the intramuscular (IM) injections of the following synthetic glucocorticoids: Dexamethasone phosphate (6 mg), Betamethasone acetate (3 mg) + Betamethasone phosphate (3 mg), or Betamethasone phosphate (6 mg). Betamethasone was only half of the single antenatal betamethasone dose. The limit of the synthetic steroid (∼1 ng/ml) known to suppress the endogenous cortisol synthesis is shown by dashed line. As a result of the slow release of intramuscular betamethasone acetate, its concentration in plasma exceeds 1 mg/ml even 4 days after the injection. This is likely to take place in pregnant mothers and since the synthetic steroid passes to the fetal blood, suppression of cortisol synthesis of the fetus and the newborn infant shortly after the birth is anticipated. The data shown is from the study by Jobe et al. (59, 141). Copyright from Springer Nature, and from Wiley Periodicals, Inc.

According to Jobe et al., the slow release of i.m. BM-acetate leads to persistence of the drug in circulation (59). On the other hand, the release of i.m. BM- or DX-phosphate to blood proceeds within less than 5 or 2 h, respectively, and both have high peak concentrations. BM and DX, unlike cortisol, enter to the fetal blood. However, i.m.BM-acetate, constituting 50% of the BM drug, is slowly released to the blood. In addition, four days after 3 mg BM-acetate, the BM serum levels are detectable by a sensitive detection method and suppresses endogenous cortisol (Figure 1). Since the BM-acetate treatment dosage of 6 mg × 2 q. 24 h i.m. (i.e., half of the ACS dosage) very slowly enters to the maternal blood and spreads the fetal compartment, BM-acetate may persist in the fetal blood for several days and contribute to the suppression of endogenous cortisol secretion shortly after premature birth (59). On the other hand, for the induction of fetal maturity, a sustained glucocorticoid exposure of the fetus appears necessary.

The beneficial effect of ACS after threatened premature birth was not evident in a cluster-randomized trial within six moderate-to-low income countries (Argentina, Guatemala, India, Kenya, Pakistan, and Zambia) (60). ACS treatment was associated with significant decrease in the survival rate of infants and increase in infections. However, another randomized trial in middle-to-low economy countries revealed that i.m. DX increased the neonatal and perinatal survival rates without detectable adverse effects. ACS was given to mothers with well-defined length of pregnancy, imminent preterm birth, and no evidence of intrauterine infection (61).

At present, the optimal ACS drug formulation, dosage, and time of administration remain to be defined. Pharmacodynamic reevaluation, including pharmacokinetic studies, model studies of efficacy, and safety, are needed. An ACS drug formulation with rather fast adsorption rate and a dosage allowing moderate and continuous glucocorticoid exposure of the fetus for not longer than 3–4 days appears desirable. Another challenge is to define biomarkers that accurately predict the duration of the remaining preterm pregnancy and minimize the unnecessary ACS treatments. New randomized trials using a modified ACS-formulation with long-term follow-up appears to be indicated.

### Surfactant Therapy

Surfactant supplementation, together with a combination of new perinatal management practices, has virtually eliminated RDS deaths in infants born around 26 weeks or later and allowed the survival of preemies who are multiple weeks below the survival limit of 28 weeks gestation during 1980s. Nowadays, a large majority of ELGA infants survive in advanced perinatal centers. However, many of them still die of generalized cardiorespiratory failure during the first week despite maximal cardiopulmonary treatment, including surfactant therapy, advanced ventilation practice, and hemodynamic drugs. Insufficient gas exchange, caused by the small surface of the respiratory bronchioles, deficient capillary network, and the long distance between terminal airspaces and capillaries, are factors limiting the survival of extremely immature infants.

#### Surfactant Administration Without Intubation

A significant advance in management was extubation to continuous distending pressure soon after the surfactant administration (INtubation-SURfactant-Extubation, INSURE) (62) rather than traditional mechanical ventilation. The next innovation was the less invasive surfactant administration (LISA) (17, 63) and similar techniques, i.e., minimally invasive surfactant (MIST) (64). A thin catheter is introduced into the trachea, followed by application of surfactant and removal of catheter, while non-invasive ventilation continues. Meta-analysis of a total of 2,164 infants in 16 trials comparing the outcome of LISA *vs.* INSURE or LISA vs. non-invasive continuous airway pressure revealed that LISA was associated with the lowered likelihood of composite death or BPD at 36 weeks’ postmenstrual age (65). According to a recent multicenter randomized trial comparison of MIST treatment to placebo treatment of 488 infants born during 25–28 postconceptional (PC) weeks, MIST is associated with a decrease in the incidence of BPD (19). These results leave some issues open, particularly on how early surfactant may be administered and how effectively mechanical ventilation is avoided. Surfactant administration *via* thin catheter may require prophylactic pain treatment. Finally, these techniques require sufficient efforts of spontaneous ventilation, excluding some extremely immature and sick surfactant recipients.

#### Timing of Surfactant Therapy After Birth?

According to meta-analysis of randomized trials, prophylactic surfactant treatment shortly after birth had no beneficial effects on survival without BPD compared to early selective treatment (13). Trials in the 1990s preferred prophylactic surfactant, but the definition of BPD back then is different from the new applied BPD definition in the 2000s. The individualized early surfactant treatment in RDS rather than prophylactic surfactant after extremely premature birth became dominant. Prophylactic surfactant treatment has also changed, i.e., up to 1 h after birth rather than very shortly after birth, while the first rescue surfactant during established respiratory distress is given within 2–6 h. In trials during the 2000s, the infants were often treated with continuous distending pressures, whereas during the 1990s, less-developed mechanical ventilation was used and therefore likely influenced the outcome.

Direct prophylactic surfactant administration to the fetal lung fluid after delivery and before the onset of air breathing offered a striking benefit in experimental prophylactic surfactant treatment of very premature sheep compared to early surfactant administration within less than 1 h after birth in maximally ventilated animals (66). This observation may no longer be valid since the old ventilation technique was used. In addition, it is difficult to deliver surfactant to an immature infant before the first breath. Even a few breaths within the few minutes after birth deteriorated the pressure-volume curves of animals (67). Currently, non-invasive ventilation is dominant, but mechanical ventilation is still required for many extremely preterm infants. The respirators and the management techniques have been extensively developed since the early experimental trials (68, 69). High-frequency ventilation techniques are preferred for the treatment of stiff lungs (70).

The delayed cord clamping has been recently re-introduced in management of preterm infants at birth (71). The oxygen is delivered from the mother through the functionally intact placenta for several minutes after birth. During this grace period, administration of surfactant to the lung liquid may be possible. The pain associated with the application of surfactant can be tolerated because the endorphin levels may be high at birth (72). In case an acute invasive treatment is required during the first minutes after premature birth, a pharmacological pain treatment for the mother using a drug with free access to the fetus maybe considered.

#### Quality and Indications of Exogenous Surfactant

In a study reported in 1948, a detergent that lowered the surface tension was introduced to airways at birth by Gruenwald (73). After the discoveries of the function of alveolar surfactant by Pattle and Clements in 1955/1956, Mary Ellen Avery reported in 1959 that the lung effluent in hyaline membrane disease did not contain surface active material. Soon after that, the main surfactant component was identified and the supplementation therapies followed: first, using dipalmitoyl phosphatidylcholine (DPPC) and thereafter with mixture of DPPC and another surfactant component, phosphatidylglycerol, or spreading agent, hexadecanol. In 1980, Fujiwara reported the acute beneficial surfactant effect in RDS using an extract of bovine surfactant (73). Natural human surfactant, containing all surfactant proteins, including species-specific SP-A, a collectin-family glycoprotein, was an investigational drug in clinical trials from 1981 to 1991 (74–76). However, domestic animals were proven to be a stable and adequate source of commercial surfactant for treatment of RDS. As a result, animal surfactants containing characteristic lipids and highly conserved hydrophobic SP-B and SP-C devoid of potentially immunogenic heterologous collectins became dominant surfactant drugs by 2000s (77).

The synthetic surfactants containing phospholipids and peptides mimicking SP-B and/or SP-C have been mostly used in trials (78, 79). The first commercial KL_4_-peptide-containing synthetic surfactant aims to capture the surface-adsorption-promoting properties of SP-B (80, 81). The currently available peptide-containing synthetic surfactants were nearly as beneficial as animal surfactants (82). Effective natural surfactant is less viscous, resistant against surfactant inhibitors, has very fast surface adsorption, and is effective in treatment of experimental lung immaturity. A commercial porcine surfactant, Curosurf, does not have disturbing viscosity despite exceptionally high concentration (80 mg/ml), allowing high dosage treatment using a near-optimal carrier volume. Surfactant is added as one to few boluses to the airways. Despite improved techniques, nebulized surfactants are less effectively delivered to terminal airways than the bolus of surfactant.

Will the quality of surfactant drugs continue to develop? Natural surfactant contains collectins, principally SP-A and SP-D, that are highly species-specific glycoprotein polymers. They influence the immune properties of the complex and destroy respiratory viruses (83, 84). In addition, collectins interact with macrophages and increase the phagocytosis of Mycobacteria and Streptococcus pneumonia by increasing the cell surface localization of phagocytotic receptors (85). Both the low SP-A content in the tracheal aspirate (86) and Ureaplasma pneumonia (87) are associated with the risk of BPD. Collectins may provide interesting properties to commercial surfactants (88). According to a recent plan, recombinant human SP-D is intratracheally supplemented to infants as nanoparticles, providing a sustainable release (89). The aim is to first investigate the scientific validity of this approach and eventually study whether a surfactant containing human collectin further decreases the risk of BPD, as suggested in original trials using human surfactant (75).

Budesonide, added to a commercial surfactant, has increased the survival of immature infants without BPD. A somewhat similar effect was obtained when budesonide was nebulized to the airways or when a low-dose of parenteral hydrocortisone was given during the first week (6). The indications, dosage, the route, and the quality of corticosteroid administrated soon after extremely premature births remain controversial. However, surfactant, as a topical carrier of drugs for RDS and other pulmonary diseases, may prove to be useful (84, 88, 90).

## Management of Transitional Circulation

Transitional circulation of immature newborns is often complicated by hemodynamically significant PDA with left-to-right shunt or, more rarely, with early PPHN with right-to-left shunt. A leaky pulmonary vascular bed and the limited cardiac function further predispose immature newborns to severe lung edema or ischemia. Early ultrasound diagnostics, excluding cardiac malformations and the follow-up of developing hemodynamics during the postnatal course, is required. The reader is referred to the current treatment recommendations for the management of RDS (6) and the advanced cardiopulmonary management protocols (91). The resent brief review focuses on the controversy of the indications, the quality of the prostaglandin (PG) synthesis inhibitor drugs used for the PDA closure, and the indications of iNO during abnormal early neonatal circulatory transition.

### Is Prophylactic Paracetamol Useful in Management of Immature Infants?

During the early days of the discovery of the medical closure of PDA, cardiorespiratory distress due to PDA was considered by some to be a significant factor causing RDS (92). At present, individualized treatment of PDA, mostly after the first week, is recommended (6). According to meta-analyses of clinical trials, medical closure of PDA does not decrease the risk of serious neonatal morbidities, such as BPD, IVH, or NEC, and is not associated with any long-term benefits (93). The consensus statements in the United States and Europe favor selective medical treatment of symptomatic PDA (6, 94). Ligation using open surgery or intravascular device is indicated when the medical treatment of symptomatic PDA fails or is contraindicated. PDA surgery may rarely take place during the first week. In addition, detailed diagnosis and treatment protocols have improved the management of PDA (95).

Complex mechanisms regulate the patency, contraction, and closure of PDA, and any medical treatment is expected to fail at times. The advances in the diagnostics of hemodynamic stress and the understanding of the pathogenetic role of a large left-to-right shunt have improved the diagnostics and accuracy of the indications of medical PDA closure (95). This may lead to more favorable results for the management of PDA. Cohort studies demonstrate the benefits of early selective treatment of PDA during the first neonatal week (96, 97).

Spontaneous closure of PDA takes place soon after birth in some extremely immature infants, and this is an argument against the prophylaxis of PDA closure. The side effects of non-steroidal anti-inflammatory agents (NSAIDs), indomethacin and ibuprofen, are prominent soon after birth. They cause dose-dependent and non-specific inhibition of prostaglandin, thromboxane, and prostacyclin synthesis (98, 99). Besides the risk of bleeding, indomethacin use is particularly associated with renal insufficiency. Bowel perforations have been particularly associated with ibuprofen. NSAIDs also potentially complicate the PDA closure, as they prevent adherence-promoting thrombosis within the intima of the constricted PDA (100).

After the first evidence of paracetamol (acetaminophen)-induced closure of PDA (101), multiple studies have affirmed this finding (93, 102). Paracetamol is a specific inhibitor of the peroxidase component of the PG synthase complex. It has been suggested that the PG synthase complex, providing substrate for prostaglandin E (PGE)-synthesis, contains low quantities of arachidonate and peroxide substrates, allowing dose-dependent inhibition of PGE synthesis by paracetamol (98). The premature ductus ring is occupied with EP4 receptors that are activated by PGE, maintaining PDA under conditions of hypoxia (103). Paracetamol does not remarkably inhibit the synthesis of thromboxane or prostacyclin (98). Excess of peroxides during an inflammatory storm is expected to decrease the efficacy of paracetamol in the inhibition of PGE synthesis.

Paracetamol has a narrow therapeutic window, and the serious hepatic toxicity is a rather common self-inflicted event (98). The polymorphic CYP450 enzyme activities are very low in infants. Therefore, a very small quantity of the toxic metabolite is formed (104, 105). The fetus is exposed to toxic paracetamol metabolites from the mother, and the maternal use of paracetamol during pregnancy has been associated with increased risk of several diseases in childhood, including asthma, autism spectrum disorders, and ADHD (106, 107). In a hospital register-based follow-up study until the age of five, the newborn infants receiving i.v. paracetamol had similar risk patterns of asthma or neuropsychiatric disorders than the non-exposed sick newborn (108). An observational study revealed a decrease in opioid use among VLGA children after the introduction of i.v. paracetamol for pain treatment (109). Currently, paracetamol is the preferred treatment of symptomatic PDA in thrombocytopenia, bleeding disorders, or when NSAIDs fail to close PDA. The placebo-controlled blinded trials of paracetamol treatment, starting during the first day of life, increased the closure rate of PDA (110). The effect on PDA closure was striking when a low paracetamol dosage was given within 6 h after birth of infants born before 27 weeks of gestation (111). The phase 2 trials using paracetamol and other trials mostly after the 1st week revealed no adverse effects. In one early trial, paracetamol tended to decrease the early hemodynamic stress due to PDA closure (112), and it decreased the levels of C-reactive proteins, suggesting moderation of inflammation (113).

The studies on paracetamol for the prophylactic closure of PDA justify further multicenter randomized trials with long term follow-up. The multicenter TREOCAPA trial (Prophylactic treatment of the ductus arteriosus in preterm infants by acetaminophen) is currently recruiting infants, born between weeks 23 to 28 of pregnancy (114). The i.v. paracetamol dosage resembles what was used previously: loading at 20 mg/kg and maintenance at 7.5 mg/kg × 4 for five days. This randomized phase 3 trial includes a nested pharmacodynamic study. The aim of this phase 2 dose-escalation trial is to study whether the increase in the dosage increases the efficacy of PDA closure without affecting safety. This trial only includes infants born at 23–26 weeks of gestation since these infants had only a barely detectable response to a standard dosage of paracetamol (115).

Left-to-right shunt across PDA and its complications are suggested to increase the risk of BPD. Whether the early paracetamol treatment increases survival without serious adverse effects, such as Grade 2-3 BPD, grade 3–4 IVH, or stage 2–3 NEC, remains to be shown. In addition, long term follow-up of the participants of the TREOCAPA trial is required to demonstrate the lack of adverse effects before prophylactic paracetamol may become an accepted therapy.

### Is Inhaled NO Indicated in Complicated RDS of Very Premature Infants?

**FIGURE 2:**
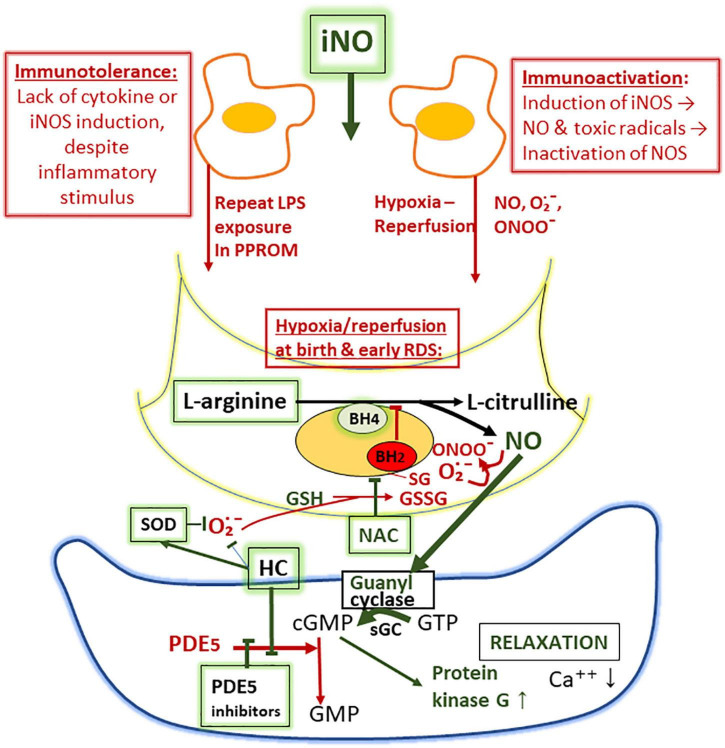
Proposed mechanisms of suppression of nitric oxide (NO) synthesis in the lung as a sketch depicting macrophage **(above)**, endothelial cell **(middle)**, and smooth muscle cell **(below)**. The lack of NO synthesis is proposed to be due to (i) failure of iNOS synthesis due to immunotolerance, associated with inflammatory insults by prolonged rupture of fetal membranes and (ii) oxidant-induced inactivation of endothelial NO synthase (eNOS) involving glutathionylation, resulting in diversion of the NO synthetase (NOS)-catalyzed NO production to superoxide (O_2_^∙−^) production (uncoupling). Superoxide converts NO to toxic peroxynitrite (ONOO^–^). Tetrahydrobiopterin (BH4), an essential co-factor of eNOS, may also be oxidized to BH2 upon inactivation of eNOS during ischemia-reperfusion injury. Phosphodiesterase-5 (PDE5) inhibitors (sildenafil), hydrocortisone (HC; inhibits PDE5 synthesis and the formation of superoxide), superoxide dismutase (SOD; breaks down superoxide), and N-acetylcysteine (NAC; source of cysteine and reduced glutathione) and L-arginine (substrate of NOS) are augmenting the formation and the activity of NO.

Onset of pulmonary oxygen uptake associates with an acute decrease of high fetal pulmonary arterial resistance shortly after birth. Several regulators, including bradykinin, prostacyclin, and NO, decrease the fetal contractile state of the pulmonary resistance vessels. The NO synthesis is catalyzed by endothelial NO synthetase (eNOS). It activates membrane-bound guanylate cyclase in smooth muscle cells of the resistance vessels, forming the potent vasodilator cyclic GMP (cGMP) (Figure 2). The half-life of endogenous NO is a fraction of seconds as this molecular chameleon reacts with a number of heme-proteins and other reactive molecules (116).

In VLGA infants, dysfunctional cardiorespiratory adaptation may manifest as early severe PPHN soon after birth (117). It presents as right-to-left circulatory shunt across the fetal channels of an anatomically normal heart and as severe hypoxia requiring high fraction of inspired oxygen (FiO_2_) despite effective ventilation. Many affected infants respond to surfactant very shortly after birth. Once they develop PPHN, they fail to respond to conventional treatments, often after a grace period for few hours or more. In previous studies, in reporting the iNO-treatment of early PPHN of VLGA infants, nearly all were born after PPROM (118, 119). At present, the population of the VLGA infants with early PPHN treated with iNO has a more diverse spectrum of antenatal complications than previously (118). Besides PPROM, antenatal complications include maternal diabetes, preeclampsia, and birth asphyxia. Despite an apparent increase in incidence of early PPHN, only 3–8% of the VLGA population is currently affected by PPHN. In most surveys of VLGA infants, iNO treatment is considerably more common (120), suggesting heterogeneity of indications for iNO therapy.

Several diseases simulate the manifestation of PPHN. In extreme structural immaturity of the lung during the canalicular stage, namely, those mostly born gestation weeks 22–23, the small pulmonary vascular bed, long alveolar-capillary diffusion pathway, and the small surface area of terminal airways severely limits the gas exchange, and PPHN-simulating condition develops right from the birth (120, 121). These infants likely fail to survive. Some other infants may present with early severe pneumonia or other acquired lung injuries (12). Furthermore, lack of urination of the fetus results in deficient amniotic fluid formation, early oligohydramnios, and lung hypoplasia. Likewise, early PPHN is frequently diagnosed as lung hypoplasia based on the small size of the lung and growth restriction during prolonged oligohydramnios due to PPROM. Amnioinfusion has been attempted for treatment of anuria and very early PPROM without evidence of benefit (122). In some cases, PPHN-like syndrome associates with metabolic defect, causing deficiency in the arginine supply for NO synthesis (Lysinuric Protein Intolerance, LPI, as an example) (123). Alveolar-capillary dysplasia (ACD) is a rare congenital fatal disease with misplaced pulmonary capillaries (117). These and other congenital alveolar dysplasia cases often have an established genetic defect (124). Deficiency in left cardiac output, associating with large right-to-left shunt through PDA, mimics the symptoms of PPHN. The most critical exclusion in early differential diagnosis of PPHN is congenital cyanotic heart disease.

Besides dependence of high supplemental oxygen, a common feature of early PPHN is remarkably low lung compliance that does not respond to surfactant therapy. These symptoms often indicate high-frequency ventilation and early onset of iNO. Soon after the onset of iNO treatment in PPHN, there is often an acute increase in lung compliance, necessitating acute adjustment of the high airway pressures, along with the dramatic decrease of O_2_ requirements to avoid excessive lung distention. The increase in lung compliance in early PPHN is faster and more pronounced than in RDS following surfactant supplementation, suggesting a different mechanism. It has been proposed that in PPHN associated with PPROM, the immune tolerance, i.e., failure of cytokine responses develops (118), mimics the immune tolerance of the immature fetal lambs after multiple LPS-treatments (125). As a result, the inflammatory NO synthase (iNOS) enzyme is not induced, and contracted small airways may fail to relax. The inhibition and uncoupling of eNOS after hypoxia-reperfusion possibly affects all NOS enzymes located within the very hypoxic lung compartments exposed to hypoxia-hyperoxia injury (126). The proposed mechanism of uncoupling of NOS is due to oxidative stress-induced superoxide (O_2_^∙−^) generation and oxidative depletion of cofactor tetrahydropteridin (BH_4_) (Figure 2). The oxidative stress also induces S-glutathionylation of NOS synthase at the critical cysteine residue that serves as a redox switch influencing the uncoupling of NO and induction of O_2_^∙−^ generation. According to experimental evidence, supplementation of BH4 or increasing the availability of intracellularly reduced glutathione (GSH) by supplementation of N-acetylcysteine (NAC) potentially increases the NO synthesis (127, 128).

A study of nitrate/nitrite levels in airway specimens from VLGA infants of mothers with severe preeclampsia revealed a consistent deficiency, indicating lack of NO generation (118). These VLGA infants had severe RDS, but were not treated with iNO. The potential explanation of the lack of NO in the airway specimens of these electively delivered infants of mothers with severe preeclampsia is severe hypoxia followed by reoxygenation injury at birth. Recently, a suppression of NO synthesis in placental endothelial cells in preeclampsia was observed (129).

During the iNO treatment, the small lung volume in PPHN expands as the contractile state decreases. The size of the lung often remarkably expands during the treatment and appears normal in size during the recovery. In most cases of early PPHN, the duration of PPROM and oligohydramnios lasted more than a week, and the fetal compression of the chest likely restricted the lung growth. Severe early neonatal respiratory insult, such as severe RDS or lung hypoplasia, are well-known risk factors of BPD. In the previous study of the 17 iNO-treated VLGA-infants, two died and nine had grade 2 BPD (118). We anticipate that the outcome may improve when the acute management practices become established in Neonatal Intensive Care Units (NICUs). The rapid adjustments in continuous distending pressure requirements and a gradual decrease of the maximum iNO concentration of 20 ppm soon after the acute response to iNO are critical components in the management. The aim to discontinue the iNO treatment within less than 24 h after the onset may not always be possible, suggesting a heterogeneity of the underlying causes of early PPHN. Very early discontinuation of iNO may result in relapse, and prolonged treatment is likely associated with toxicity (Figure 2).

In some cases, the patient fails to respond to iNO (120). The diffusion pathway of iNO may be too long due to extreme immaturity, excessive edema, insufficient distending pressures, or due to other causes of critical hypoxemia, such as acute lung rupture or congenital cardiac malformation. In our practice, iNO treatment is not attempted when an extremely immature infant (21–23 weeks of gestation) fails to respond to resuscitation. On the other hand, according to therapeutic trials, iNOS treatment has no significant beneficial effects to VLGA infants with uncomplicated respiratory distress (120, 130). The controversy on the indications and potential beneficial effects of iNO will likely continue. Besides the decrease in pulmonary vascular resistance, NO may also decrease the excessive resistance of the small airways, promote the antimicrobial defense, and maintain surfactant function. The toxic effects of iNO in experimental studies include the inactivation of surfactant and life-threatening oxidative effects of toxic free radicals and peroxynitrite that may damage DNA and proteins, including the mitochondrial respiratory chain (116). Thus, minimizing the toxicity of NO and maintaining the apparently life-saving effect of iNO on VLGA infants with PPHN is a goal.

In the placebo-controlled iNO-trials involving the VLGA-infants, the indications have ranged from mild to moderate-severe respiratory distress, and only a small minority have been identified as PPHN-like disease (131). According to meta-analysis, no increase in survival without BPD was observed, with an apparent exception of infants with African-American background. According to cohort studies, most patients with iNO treatment of early PPHN have survived, whereas the untreated historical controls almost exclusively died (118, 130).

Further clinical trials comparing iNO and alternative vasodilator treatments in early PPHN of VLGA infants are indicated. Randomized phase 2 trials involving iNO treatment of the newborn with pulmonary hypertension and another comparison drug include endothelin receptor antagonists (Bosentan) (132, 133). Inhibitors of phosphodiesterase 3A (Milrinone) (134) or of phosphodiesterase 5A (Sildenafil) (135) may be used as an adjunctive therapy or as the comparison treatment. Thus far, these trials have included term or near-term infants with PPHN. In addition, experimental evidence on HIF1α inhibition (136, 137), hydrocortisone (138), superoxide dismutase (SOD) (139), NADPH oxidase inhibitor (Apocynin) (140), and other agonists and antagonists that influence neonatal PPHN (133) have been reported. In the future, randomized trials on iNO may include dose-response, dose-escalation, and drug comparison trials. An attractive comparison or adjunctive drug would involve a supplementation that facilitates both the pulmonary vascular adaption and the alveolar stability in synergy with NO.

## Conclusion

New supplementation treatments or prophylactic treatments may eventually prove to be safe and effective for improving the less satisfactory outcome of the most immature infants. The best scenario would be a drug that may be given to the mother for prevention of extremely preterm labor and delivery and to an extremely premature newborn for early treatment of respiratory distress. Glucocorticoid supplementation is extensively used before and after birth. Critical pharmacodynamic studies are required for defining the effective and least damaging dosage. Respiratory distress in extremely preterm infants presents as a multiorgan failure that requires integrated cardiopulmonary management strategy. Less invasive surfactant treatment and non-invasive ventilation with caffeine supplementation is a preferred treatment strategy in most cases. However, it may not be successful for the most immature infants and for the rare cases of complicated and severe respiratory distress. For the most immature infants, surfactant may be given as soon as possible at birth, followed by fast and thoughtful weaning from mechanical to non-invasive ventilation. Despite this, how to further protect against injuries and promote healing and growth remains a challenge. Failure of less invasive strategy requires rapid diagnostics and modification of treatment. Establishing the preferred ventilation mode and settings, management of perfusion, avoiding excess of fluid intake, limiting excessive left-to-right shunts, and, if necessary, establishing effective pulmonary vasodilator treatment via the airways need to be included in the strategy. Rapid weaning from effective and at the same time damaging therapies is a priority. Lastly, new medications that strengthen the immature or dysfunctional defenses throughout the very premature perinatal transition, prolonging very premature pregnancies, and further decreasing respiratory distress and serious multi-organ consequences may provide the next leap in neonatal—perinatal medicine.

## Author Contributions

MH conceptualized, discussed with collaborators and wrote the manuscript. ER collected and analyzed the data and contributed to conceptualization and writing. TS proposed and discussed the topics contributed to the writing. RM contributed to the conceptualization, discussion and writing of the manuscript. All authors contributed to the article and approved the submitted version.

## Conflict of Interest

The authors declare that the research was conducted in the absence of any commercial or financial relationships that could be construed as a potential conflict of interest.

## Publisher’s Note

All claims expressed in this article are solely those of the authors and do not necessarily represent those of their affiliated organizations, or those of the publisher, the editors and the reviewers. Any product that may be evaluated in this article, or claim that may be made by its manufacturer, is not guaranteed or endorsed by the publisher.
